# Reoperative Resection of Killian-Jamieson Diverticulum Using Indigo Carmine-Assisted Endoscopic Localization: A Case Report

**DOI:** 10.7759/cureus.94942

**Published:** 2025-10-19

**Authors:** Atsushi Yoshiyama, Shinsuke Sato, Erina Nagai, Masaya Watanabe, Hideyuki Kanemoto

**Affiliations:** 1 Gastroenterological Surgery, Shizuoka General Hospital, Shizuoka, JPN

**Keywords:** endoscopic die injection, indigo carmine, intraoperative endoscopy, killian-jamieson diverticulum, reoperation, zenker’s diverticulum

## Abstract

Killian-Jamieson diverticulum is a rare cervical esophageal diverticulum that is often difficult to identify intraoperatively because of its small size and lateral location. To our knowledge, there have been no case reports requiring reoperation because a small diverticulum was difficult to identify intraoperatively. We report a case of a 44-year-old man with progressive dysphagia whose initial transcervical diverticulectomy was incomplete because the diverticular orifice could not be clearly identified. At reoperation, intraoperative endoscopic indigo carmine injection enabled precise localization and complete, safe resection. This case highlights that indigo carmine injection may serve as a simple and effective adjunct to facilitate accurate identification and successful resection of small or obscure esophageal diverticula.

## Introduction

Killian-Jamieson diverticulum (KJD) is a rare type of esophageal diverticulum. Among cervical esophageal diverticula, the most common is Zenker’s diverticulum (ZD), which arises from a muscular gap in the posterior wall located above the cricopharyngeal muscle and below the inferior pharyngeal constrictor muscle. In contrast, KJD is the second most common cervical esophageal diverticulum, originating from the anterolateral wall between the transverse portion of the cricopharyngeal muscle and the lateral aspect of the longitudinal esophageal muscle [1-3]. KJD is considered extremely rare and has been reported far less frequently than ZD in the literature [4,5]. Patients with symptomatic KJD may present with dysphagia, globus sensation, halitosis, regurgitation, cough, or aspiration, and both surgical and endoscopic treatments have been described [4-6]. However, diagnostic challenges remain; there are reports of cases initially misdiagnosed as ZD but later found to be KJD intraoperatively [2]. To our knowledge, no published reports have specifically described cases in which small KJDs were difficult to localize intraoperatively, resulting in incomplete resection and necessitating reoperation. Therefore, strategies to ensure accurate intraoperative localization may be required when managing these rare and anatomically obscure diverticula. Here, we report a case in which the initial transcervical diverticulectomy was unsuccessful due to inadequate anatomic identification, whereas reoperation with intraoperative endoscopy and indigo carmine injection enabled accurate localization and complete resection.

## Case presentation

**Figure 1:**
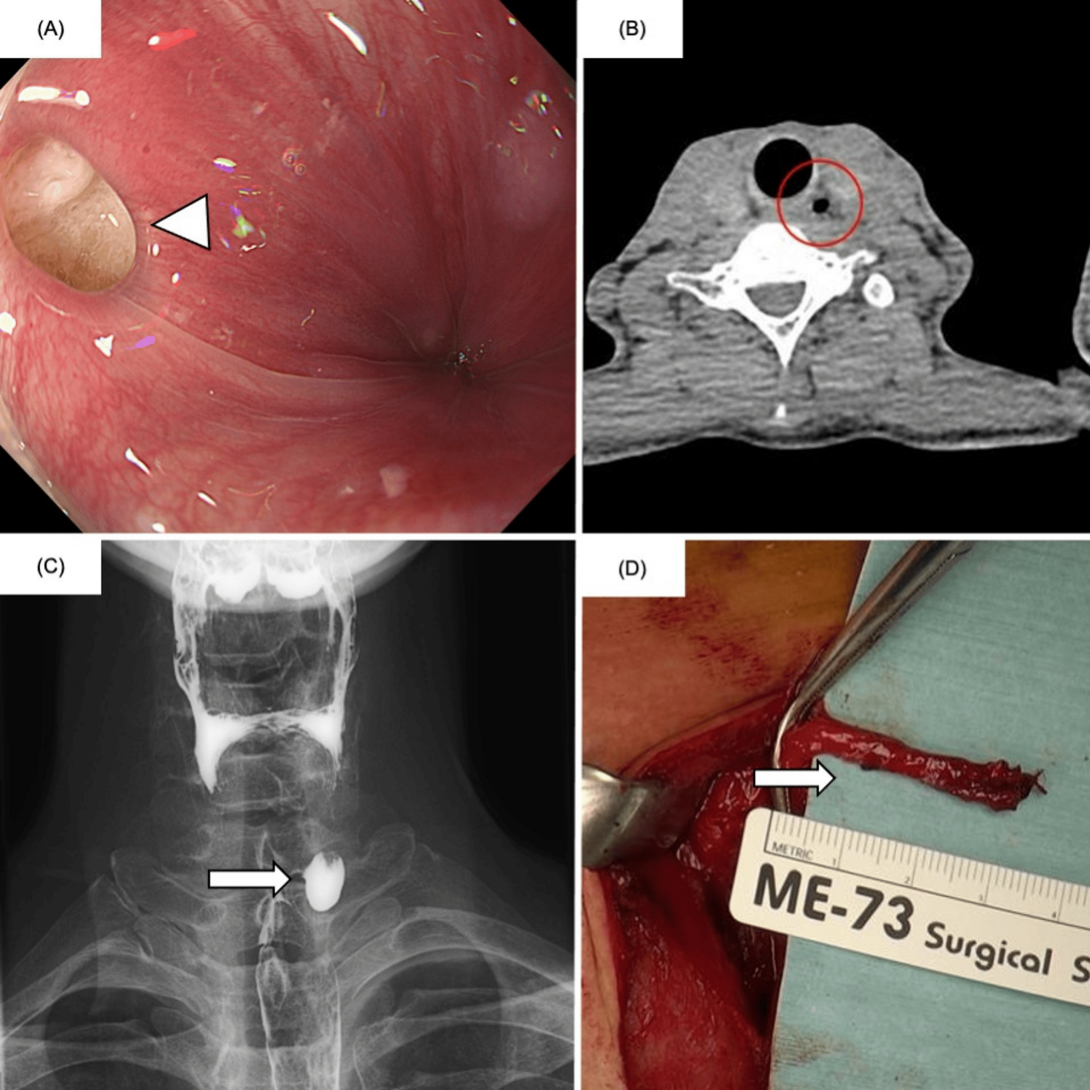
Preoperative imaging and resected specimen from the first surgery. (A) EGD revealed a diverticulum arising from the left wall of the cervical esophagus (white arrowhead). (B) Cervical CT demonstrated an air-filled outpouching consistent with a diverticulum on the left side of the cervical esophagus (red circle). (C) Barium esophagogram confirmed a 1.5-cm diverticulum on the left side of the cervical esophagus (white arrow). (D) Intraoperative view of the structure identified as the diverticulum during the first operation (white arrow). EGD, esophagogastroduodenoscopy; CT, computed tomography

A 44-year-old man presented to our hospital with a six-month history of globus sensation. Esophagogastroduodenoscopy revealed a diverticulum on the left cervical esophageal wall 18 cm from the upper incisors (Figure 1A). Cervical computed tomography showed an air-containing diverticulum on the left side of the cervical esophagus (Figure 1B). A barium esophagogram confirmed a 1.5-cm diverticulum in the same location, which was relatively small (Figure 1C). KJD was diagnosed based on these imaging findings. Treatment options, including transcervical diverticulectomy and an endoscopic approach, were discussed, and the patient elected to undergo surgical excision.

**Figure 2:**
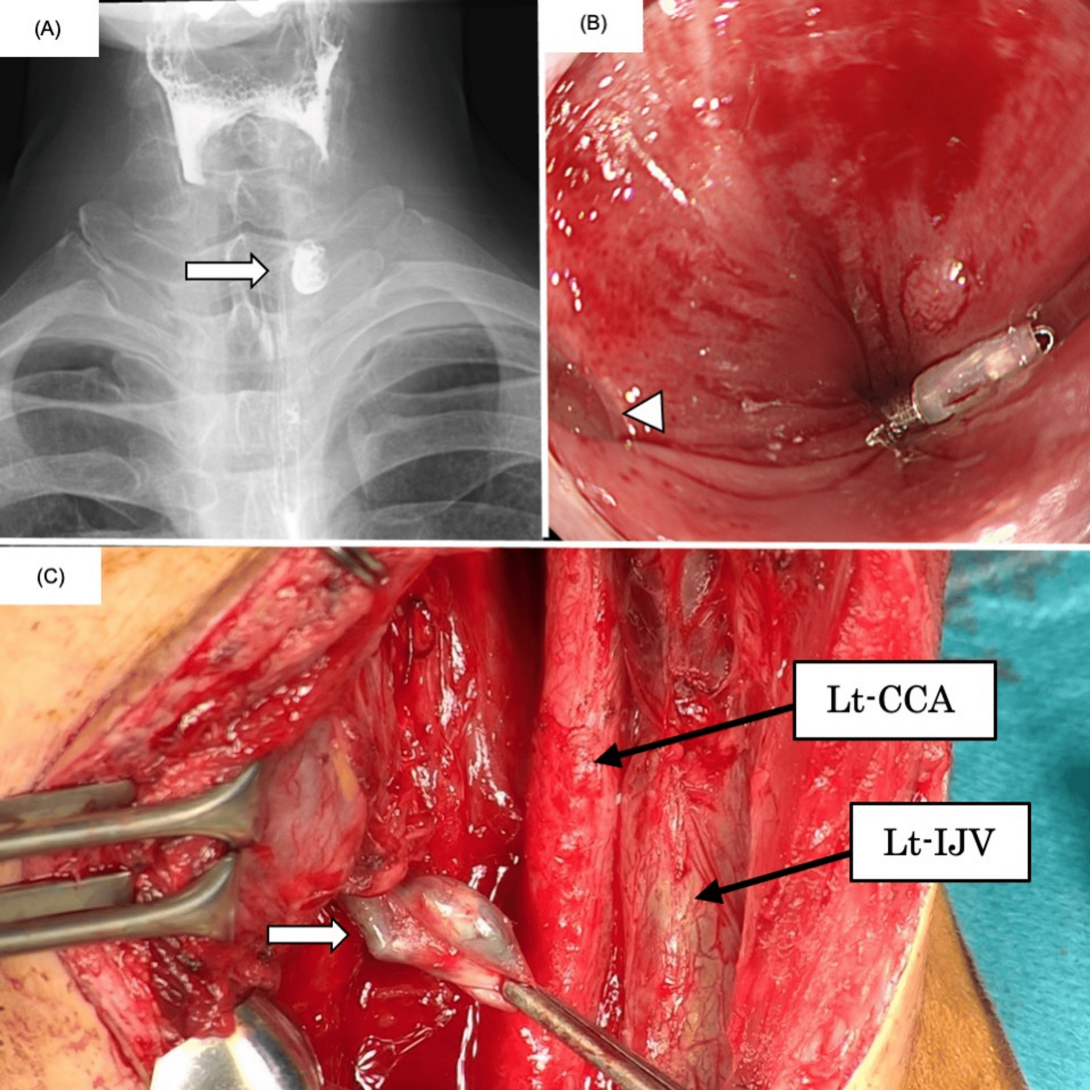
Postoperative and reoperative findings. (A) Barium esophagogram performed after the first surgery demonstrated persistence of the diverticulum (white arrow). (B) Before reoperation, a marking clip was placed endoscopically near the diverticular orifice (white arrowhead). (C) During reoperation, the diverticulum was clearly identified by endoscopic indigo carmine injection into the pouch (white arrow). CCA, common carotid artery; IJV, internal jugular vein

Transcervical diverticulectomy was performed under general anesthesia. The patient was placed in the supine position with neck extension, and a collar incision was made in the left anterior neck, 1 cm above the clavicle, extending from the midline toward the left cranial direction. After dividing the platysma, the sternocleidomastoid muscle and carotid sheath were retracted laterally to expose the esophageal wall. Intraoperative endoscopic visualization of the cervical esophageal lumen was challenging, and the diverticular orifice appeared less distinct than during preoperative endoscopy. A structure in the cervical surgical field judged to be the diverticulum was ligated and resected (Figure 1D). Intraoperative nerve monitoring confirmed preservation of the left recurrent laryngeal nerve (RLN). The operative time was 117 minutes with an estimated blood loss of 30 mL. The postoperative course was generally uneventful; however, on postoperative day seven, barium esophagography revealed persistence of the preoperatively identified diverticulum (Figure 2A). Histopathologic examination showed that the resected specimen consisted only of periesophageal soft tissue without any diverticular component. Based on these findings, the patient agreed to undergo reoperation.

Before reoperation, a marking clip was placed approximately 5 mm distal to the diverticulum during preoperative endoscopy (Figure 2B). The reoperation was performed with the same patient positioning as in the initial surgery. Before the cervical incision, an otolaryngologist performed direct laryngoscopy, but the diverticular orifice could not be visualized. A gastroenterologist subsequently performed an endoscopy, identified the diverticular orifice using the marking clip as a guide, and inserted a contrast tube into the diverticulum. The cervical incision was extended by approximately 2 cm compared with the first surgery, and the left esophageal wall was exposed using the same approach. Indigo carmine was injected through the contrast tube, clearly revealing the diverticulum located approximately 2 cm caudal to the previous resection site on the anterior wall (Figure 2C). Dissection of the esophageal adventitia exposed a sac-like diverticulum, which was then ligated and resected. The operative time was 80 minutes with an estimated blood loss of 50 mL.

Histopathologic examination revealed a pseudodiverticulum lacking the proper muscularis layer and showing inflammatory cell infiltration. No evidence of malignancy was observed.

At six months postoperatively, the patient remained asymptomatic, and esophagogastroduodenoscopy revealed no evidence of recurrence or stricture.

## Discussion

**Figure 3:**
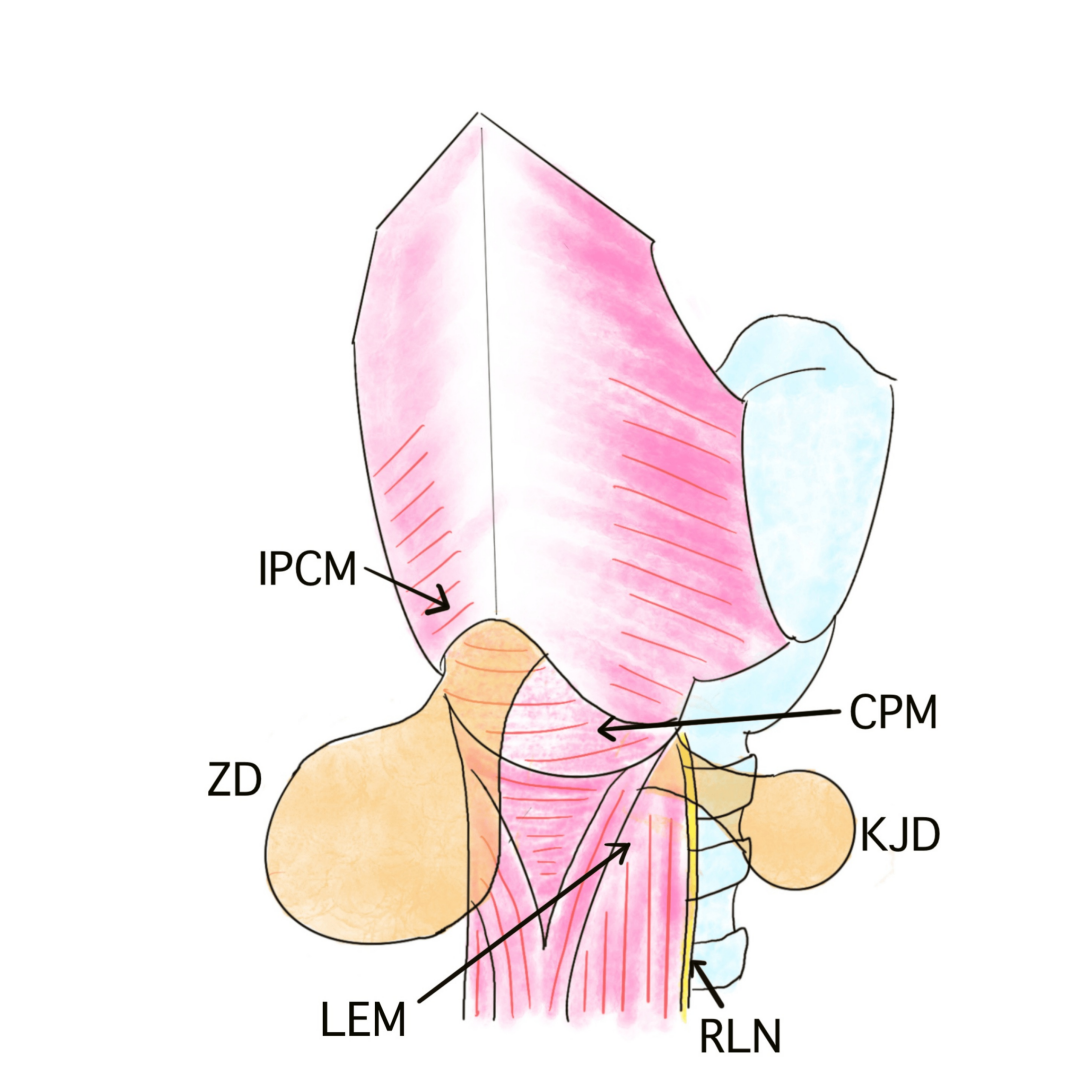
Schematic illustration of ZD and KJD. Their respective anatomical origins are highlighted. KJD, Killian-Jamieson diverticulum; ZD, Zenker's diverticulum; RLN, recurrent laryngeal nerve; IPCM, inferior pharyngeal constrictor muscle; CPM, cricopharyngeus muscle; LEM, longitudinal esophageal muscle Image Credits: Atsushi Yoshiyama

KJD is a rare cervical esophageal diverticulum that arises from the anterolateral wall of the proximal esophagus just below the cricopharyngeal muscle, whereas the more common ZD originates from the posterior wall above the cricopharyngeus (Figure 3). Because of its lateral location, KJD lies in close proximity to the RLN as it enters the larynx [1-3], making surgical dissection particularly challenging and necessitating meticulous identification and preservation of the nerve [3]. Radiologically, KJDs are usually smaller than ZDs and are often detected incidentally. Clinical symptoms such as dysphagia, cough, and throat discomfort are similar to those of ZD. Both transcervical diverticulectomy and endoscopic resection have been reported as treatment options for KJD. Endoscopic treatment offers the advantages of being less invasive and shortening hospital stay, but carries a higher theoretical risk of RLN injury because the nerve cannot be directly visualized during the procedure [7]. In contrast, transcervical diverticulectomy allows direct visualization and preservation of the RLN, providing superior safety and curative potential [3,7,8]. The use of intraoperative neuromonitoring can further enhance safety; for instance, Ataka et al. safely resected a 3 cm KJD using intermittent RLN stimulation, achieving an uneventful recovery with intact vocal cord function [3]. A comprehensive review identified 68 reported cases of KJD, of which 32 underwent surgical resection and 22 were performed via a transcervical approach. The mean diverticular size was 3.8 cm in the transcervical group versus 2.8 cm in the endoscopic group, suggesting that larger or anatomically complex diverticula are more likely to be treated surgically. Recurrence occurred only after endoscopic treatment, supporting the notion that transcervical diverticulectomy provides better long-term efficacy [9].

The present case required reoperation for KJD, an exceptionally rare event. The initial surgery failed to completely remove the diverticulum, likely because of its very small size (approximately 1.5 cm) and the difficulty in intraoperatively localizing such a small lesion. Under general anesthesia, small pharyngoesophageal diverticula may collapse and become inconspicuous, complicating identification. The absence of adjunctive localization methods or intraoperative frozen-section examination during the first operation probably contributed to incomplete resection. During reoperation, we overcame these limitations by combining intraoperative endoscopy with indigo carmine dye injection. To our knowledge, this represents the first reported use of indigo carmine injection in KJD surgery. The dye effectively colored and distended the diverticular sac, allowing clear visualization of its location and extent despite surrounding scar tissue from the previous operation. This approach enabled complete excision of the remnant diverticulum while preserving the RLN.

Intraoperative dye marking has been reported to be a valuable adjunct in other reoperative or anatomically challenging scenarios. Zhang et al. described a recurrent tracheoesophageal fistula after repair of esophageal atresia, in which indocyanine green (ICG) was endoscopically instilled into the fistula tract, providing real-time fluorescence guidance during thoracoscopy [10]. This technique allowed precise delineation of the fistula and minimized blind dissection through scarred mediastinal planes, thereby reducing operative time and tissue trauma. Similarly, visible dyes such as indigo carmine and methylene blue, as well as fluorescent agents such as ICG, can be highly effective when anatomical landmarks are obscured by prior surgery or when the target lesion is too small to be easily identified.

In summary, this case underscores several important considerations in the management of KJD. Because of its proximity to the RLN, KJD poses significant technical challenges, and small diverticula may be particularly difficult to identify intraoperatively, resulting in incomplete resection or recurrence. Reoperation for KJD is extremely rare and requires careful preoperative planning and intraoperative precision. The use of indigo carmine dye injection represents a simple, safe, and effective adjunct for accurate localization of small diverticula and may help prevent incomplete excision. Furthermore, this technique has potential applicability in other reoperative or anatomically complex surgical settings where intraoperative identification of small lesions is challenging.

## Conclusions

This case of KJD requiring reoperation underscores the importance of accurate intraoperative localization, particularly when dealing with small or inconspicuous diverticula. In this patient, endoscopic indigo carmine injection facilitated safe and complete resection by clearly delineating the diverticulum. Because no previous reports have described reoperation for KJD, such procedures should be recognized as technically demanding. Dense scar tissue and distorted anatomy can obscure the diverticulum, making meticulous preoperative planning and flexible intraoperative judgment indispensable. Moreover, close collaboration among surgeons, otolaryngologists, and endoscopists is essential for achieving favorable outcomes. The experience gained from this case may help guide clinical management in similarly rare or technically challenging situations.
